# Sexual Fate Reprogramming in the Steroid-Induced Bi-Directional Sex Change in the Protogynous Orange-Spotted Grouper, *Epinephelus coioides*

**DOI:** 10.1371/journal.pone.0145438

**Published:** 2015-12-29

**Authors:** Guan-Chung Wu, Wei-Guan Tey, Hau-Wen Li, Ching-Fong Chang

**Affiliations:** 1 Department of Aquaculture, National Taiwan Ocean University, Keelung 20224, Taiwan; 2 Center of Excellence for the Oceans, National Taiwan Ocean University, Keelung 20224, Taiwan; National Cheng Kung University, TAIWAN

## Abstract

Androgen administration has been widely used for masculinization in fish. The mechanism of the sex change in sexual fate regulation is not clear. Oral administration or pellet implantation was applied. We orally applied an aromatase inhibitor (AI, to decrease estrogen levels) and 17α-methyltestosterone (MT, to increase androgen levels) to induce masculinization to clarify the mechanism of the sex change in the protogynous orange-spotted grouper. After 3 mo of AI/MT administration, male characteristics were observed in the female-to-male sex change fish. These male characteristics included increased plasma 11-ketotestosterone (11-KT), decreased estradiol (E2) levels, increased male-related gene (*dmrt1*, *sox9*, and *cyp11b2*) expression, and decreased female-related gene (*figla*, *foxl2*, and *cyp19a1a*) expression. However, the reduced male characteristics and male-to-female sex change occurred after AI/MT-termination in the AI- and MT-induced maleness. Furthermore, the MT-induced oocyte-depleted follicle cells (from MT-implantation) had increased proliferating activity, and the sexual fate in a portion of female gonadal soma cells was altered to male function during the female-to-male sex change. In contrast, the gonadal soma cells were not proliferative during the early process of the male-to-female sex change. Additionally, the male gonadal soma cells did not alter to female function during the male-to-female sex change in the AI/MT-terminated fish. After MT termination in the male-to-female sex-changed fish, the differentiated male germ cells showed increased proliferating activities together with dormancy and did not show characteristics of both sexes in the early germ cells. In conclusion, these findings indicate for the first time in a single species that the mechanism involved in the replacement of soma cells is different between the female-to-male and male-to-female sex change processes in grouper. These results also demonstrate that sexual fate determination (secondary sex determination) is regulated by endogenous sex steroid levels.

## Introduction

Many fish species are responsive to sex steroid induction of sexual determination and development. Androgen and estrogen have been used to induce masculinization and feminization in fish, respectively [1, 2]. In addition, decreased estrogen levels (to block aromatase activity by aromatase inhibitor) also have resulted in masculinization [3]. However, unlike gonochoristic fish, in which the sex is fixed after gonadal differentiation, sexes are flexible in hermaphroditic fish. A sex change is predicted to occur at the age when the fecundity fain curves of the sexes intersect [4]. In hermaphroditic fish, increased plasma estrogen levels have also been considered important for the maintenance of femaleness (protogyny) and male-to-female sex change (protandry) [3, 5]. However, increased plasma estrogen levels result in ectopic females in adult males, and low estrogen levels also result in ectopic males in adult females in gonochoristic fish [6, 7]. Thus, ectopic germ cells and their surrounding cells are induced by the abnormal plasma levels of sex steroids. Conversely, germ cell-deficient fish typically develop as phenotypic males in medaka [8] and zebrafish [9]. In contrast, loach fish [10], goldfish [11], and black porgy [12], which lack germ cells, could develop as either phenotypic sex. Furthermore, in black porgy, ectopic oocytes localized in a regenerated testis can alter the soma sexual fate from male to a female with low estrogen levels [13]. In wrasse fish, oocyte-depleted follicle cells alter the sexual fate from female to male during the female-to-male sex change [14]. Taken together, these data suggest that the mechanism for reprogramming the surrounding cells of the oocytes is varied among species. These data also indicate that in addition to a unidirectional signal, a reciprocal cross-talk occurs between germ and somatic cells, and this cross-talk plays an important role during sexual fate alternation. However, the mechanism of sexual fate alternation in the sex change is not clear.

Protogynous groupers are cultured and provide a high quality food source and economic value in the world, especially in East Asia. However, the control of reproduction is critically important for the continued improvement of grouper brood stock. The female-to-male sex change is correlated with the size of the grouper [15, 16]. Thus, cultured groupers have the difficulty reaching the critical size (need to grow for several years) for the sex change from female to male. To solve this problem, androgens have been widely used for masculinization in grouper farms. The most commonly applied androgen is 17α-methyltestosterone (MT), which has been tested in many grouper species to induce masculinization [17–22]. However, the androgen-induced male is a transient state that reverses sex from male to female after the androgen treatment is withdrawn [18, 20, 23]. Stable maleness (no reversed sex change) is very important for the management of grouper brood stock. The characteristics of the mono-female and reversible sex change in this protogynous grouper indicate that this species is a model animal to use when investigating the mechanism of sex change, especially related to the sexual fate of gonadal soma cells.

To study the mechanism of sex change in groupers, we applied an AI/MT-induced sex change to understand the progress of the alternation of gonadal soma cells. Here, we demonstrate that AI- and MT-induced maleness is transient and that the sex change is reversed (male-to-female) after treatment withdrawal in groupers. Our data also demonstrate that the oocyte-depleted follicle cells showed increased proliferating activity and altered the sexual fate from female to male functions during the female-to-male sex change. However, the proliferating activity of male somatic cells was arrested during the male-to-female sex change. Furthermore, a dormant status with inactive early germ cells (no male and female characteristics) was observed after the MT and AI treatment were withdrawn, and sex change subsequently occurred.

## Materials and Methods

### Animals and experimental design

The experimental fish (7-mo-old female fish) were acclimated to a seawater pond environment with a natural lighting system at the National Taiwan Ocean University culture station. The mono-female grouper is an advantageous model animal for use in investigations of the regulation of sexual fate. All procedures and investigations were approved by the National Taiwan Ocean University Institutional Animal Care and Use Committee and were performed in accordance with standard guide principles.

### Experimental design

#### Fish treated with MT and AI

Before the experiment began, the gonads of differentiated juvenile female fish were collected to check the sex stage. Juvenile female fish (7-mo-old, ovary with oogonia and primary oocytes) were treated with AI/MT by oral feed administration for 3 mo or were intramuscularly implanted with an MT-pellet for 1 mo. Both treatments (oral administration for 3 mo and implantation for 1 mo) induced the fish to undergo sex change from female to male according to our previous studies [16, 17]. A diet that contained AI (1,4,6-androstatriene-3,17-dione, ATD) (n = 50; 20 mg/kg of diet; inhibited aromatase activity), MT (n = 75; 30 mg/kg of diet; increased androgen levels) or control (n = 50) was applied for 3 mo in experiment 1 and 4. Fish were kept in separated floating cages (25 fish/cage) and independent FRP tanks for different groups. Fish were fed the diet once daily and the amount of diet was more than 1% of their body weight (to make sure all fish received enough feed). To speed up the process of sex change, the intramuscular implantation with an MT pellet (100 μg MT/mg pellet; 200 mg pellet/kg of body weight) for 1 mo was conducted in the experiment 2 and 3.

#### Experiment 1: The gonadal histology, sex-related gene expressions and plasma sex steroid levels of AI/MT-induced female-to-male sex change

**Fig 1:**
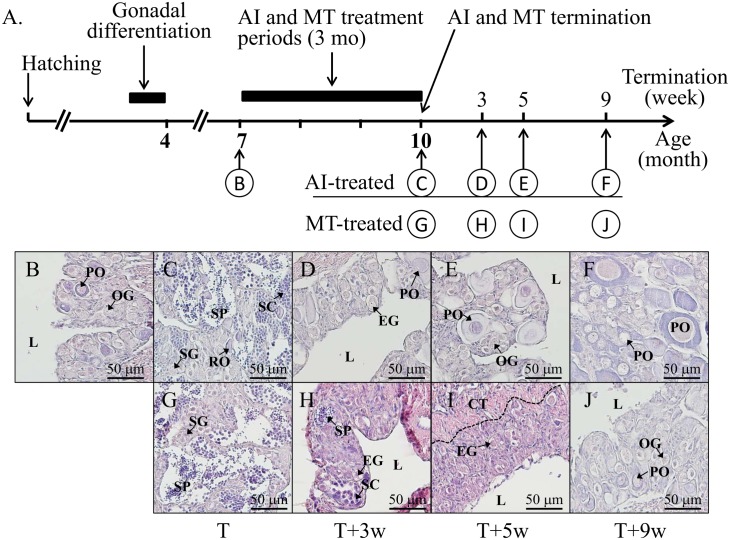
AI/MT-induced masculinization with a reversible sex change after the termination of orally administered AI/MT in orange-spotted grouper. (A) The sampling program during sexual fate alternation from female to male or vice versa, including fish collected before oral administration, and 3 mo (T, the treatment period, 7- to 10-mo-old) after AI/MT-oral administration, and after 3 wks (T+3w), 5 wks (T+5w) and 9 wks (T+9w) of AI/MT-termination. The gonadal status is shown as follows: (B) Initial control female; (C) AI-induced male with active spermatogenesis 3 mo after AI-oral administration; (D), (E), and (F) Regenerated female 3 wks, 5 wks, and 9 wks after AI-termination, respectively; (G) MT-induced male with active spermatogenesis 3 mo after MT-oral administration; (H), (I), and (J) Regenerated female after 3 wks, 5 wks, and 9 wks of MT-termination, respectively. L, central lumen; OG, oogonia; PO, primary oocyte; SG, spermatogonia; SC, spermatocyte; SZ, spermatozoa; EG, early germ cell.

The treatment of MT- and AI-administration was terminated (terminated fish) after 3 mo of oral administration. At 0, 3, 5, and 9 wks after AI/MT termination, the gonadal tissues and blood were collected to study the effects of AI/MT on the gonadal morphology, gene expression and plasma sex steroid levels (see Fig 1A).

#### Experimental 2: The cell proliferative activity of gonadal soma cells during MT-implantation (during the process of the female-to-male sex change)

To enhance the process of sex change, we conducted the sex change by applying MT-implantation. For the study of the cell survival and cell proliferation of the gonadal soma cells in the MT-implanted female-to-male sex-changed fish, the fish were implanted with an MT pellet (100 μg MT/mg pellet; 200 mg pellet/kg of body weight, n = 12) or control vehicle (n = 6) in the muscle. Furthermore, two groups were established for the experiment. Group 1): The fish were injected with Brdu (0.3 mg/g of body weight or control vehicle) on day 0 and day 3 after MT-implantation and gonadal tissues (n = 6) were collected on day 7. Group 2): The fish were injected with Brdu (0.3 mg/g of body weight or control vehicle) on day 7 and day 10 and gonadal tissues (n = 6) were collected on day 14. Gonadal tissues (collected on day 7 and day 14) were used to study the proliferating cells by immunohistochemical (IHC) staining of the Brdu-incorporated cells.

#### Experiment 3: The cell sexual fate in the reprogramming of gonadal soma cells in the MT-implanted female-to-male sex-changed fish

Furthermore, we trace the cell-sexual fate of the gonadal soma cells in the MT-induced male. MT-implantation for 30 days resulted in the sex change in the fish. For the MT-implantation (100 μg MT/mg pellet; 200 mg pellet/kg of body weight) and control vehicle, the fish (n = 6) were injected (i.p., 2 injections) with Brdu on day 7 and 10, and fish samples were collected on day 30 (n = 6 per group). The gonadal tissues (collected on day 30) were used to study the cell-sexual fate by IHC staining of the Brdu-incorporated cells.

#### Experiment 4: The sexual fate of the cell in the reprogramming of gonadal soma cells in the MT-terminated male-to-female sex-changed fish

To study the cell-sexual reprogramming of the cells in the MT-terminated male-to-female sex-changed fish, the MT diet in the MT fish was terminated after 3 mo of MT-oral administration. Three groups were further designated for the experiment. Group 1: The MT-terminated fish (day 0 is the day to start with MT-termination) were injected with Brdu (0.3 mg/g of body weight) on day 6 and day 8, and the gonad tissues were collected on day 10. Group 2: The MT-terminated fish were injected with Brdu (0.3 mg/g of body weight) on day 16 and day 18, and then the gonadal tissues were collected on day 20. Group 3: The MT-terminated fish were injected with Brdu (0.3 mg/g of body weight) on day 6 and day 8, and the gonadal tissues were collected on day 60. The gonadal tissues (collected on day 10, 20 and 60) were used to study the sexual fate of the proliferating cells by immunohistochemical (IHC) staining of the Brdu-incorporated cells.

### Gonadal histology, immunofluorescence staining and immunohistochemical staining

The fish gonads were fixed with 4% paraformaldehyde in PBS (phosphate-buffered saline). Based on the present study, 2 wks after AI/MT-termination, the gonadal status of each individual showed non-synchronous development (no clear germ cells in the anterior part, early germ cells and many spermatozoa without oocytes in the middle part, and different stages of germ cells with oocytes in the posterior part) during the male-to-female sex change (S1 Fig). Thus, the middle part of the gonad was used to identify the gonadal status and for further Immunofluorescent (IF)/immunohistochemical (IHC) staining. The gonads (5-μm-thickness sections) were treated with HistoVT One (Nacalai Tesque) to expose the antigens of the target protein. IHC staining was performed as previously described [12, 24]. For IHC staining, the section was rehydrated in a PBS and incubated with 3% H_2_O_2_ in PBS. The section was then incubated with 5% nonfat milk powder for 30 min and with anti-Brdu (1:1000 dilution; product no. MAB4072; Merk Millipore, Inc.) for overnight at 4°C. Color formation was amplified with an ABC kit (avidin-biotin, Vector) and DAB (3, 3’-diaminobenzidine, Sigma, St. Louis, MO). IHC staining was conducted with triplicate sections for each tissue (n = approximately 3~6 fish in each group).

### RNA analysis

The first-strand cDNA from gonads was used for quantitative real-time PCR (qPCR) analyses. Specific primers for *dmrt1* (doublesex and mab-3-related transcription factor 1, GenBank accession no. EF017802), *sox9* (sex determining region Y-box 9, GenBank accession no. GQ232762), *cyp11b2* (11β-hydroxylase, GenBank accession no. JQ178340), *figla* (factor in germline alpha, GenBank accession no. KP229299), *foxl2* (forkhead box l2, GenBank accession no. JQ178341), and *cyp19a1a* (aromatase gonad form, GenBank accession no. AY510711) are listed in Table 1. Gene quantification of standards, samples, and controls were conducted simultaneously by a qPCR (GeneAmp 7500 Sequence Detection System; Applied Biosystems, Foster City, CA) with SYBR green Master Mix (Applied Biosystems). The PCR specificity was confirmed by a single melting curve (at same temperature) in unknown samples and standards. The respective standard curve of log (transcript concentrations) vs CT (the calculated fractional cycle number at which the PCR-fluorescence product is detectable above a threshold) was obtained. The values detected from different amount plasmid DNA contained the fragment of target gene (10 times of series dilution) of the representative samples were parallel with the respective standard curve. The correlation of the standard curve for the genes analyses were at least -0.999. qPCR assay was done with a duplicate repeat (n = 3–8 in each group). All samples were normalized to g*lyceraldehyde-3-phosphate dehydrogenase* (*gapdh*; GenBank accession no. EU042107), and the highest value (control value) of each gene was defined as one. The *gapdh* transcripts were not significantly changed between treatments (data not shown).

**Table 1:** Oligonucleotides for specific primers used for the cloning.

Gene	Oritetation	Sequence	Characteristics
*dmrt1*	Sense	5’-GGCCCTGAGGTGATGGTGAA-3’	Male germ cell marker
	Antisense	5’-CGGGGATCGTCTCTCCACAG-3’	
*sox9*	Sense	5’-GGGGCCTTACTGTGTTGC-3’	Sertoli cell marker
	Antisense	5’-GCTCCAGAGGCACCAATG-3’	
*cyp11b2*	Sense	5’-AGACACGGCAGCACAGCAAG-3’	11-KT synthesis
	Antisense	5’-CAGCCGCACTCATCATCACC-3’	
*figla*	Sense	5’-AACGCCAAGGAGAGACTGAGAA-3’	Oocyte marker
	Antisense	5’-GGCACCATGCGCTTCAA-3’	
*foxl2*	Sense	5’-CAGGTAGCCATAGCCGTCTTCT-3’	Follicle cell marker
	Antisense	5’-CCTCCACCGACGCACTTC-3’	
*cyp19a1a*	Sense	5’-CACCAGAGGCACAAGACAGC-3’	E2 synthesis
	Antisense	5’-CCTGCTCCATGTCTCTCCTC-3’	

### Cell proliferating assay and cell tracing

Brdu incorporation into gonadal cells was used to analyze the proliferating activities and cell tracing [25, 26]. The fish were injected (intraperitoneal injection; i.p.) with Brdu (0.3 mg/g of body weight) prior to sampling. Anti-Brdu (1:1000 dilution; product no. MAB4072; Merk Millipore, Inc.) was used for IHC to identify the proliferating cells during the treatment period. Furthermore, anti-Brdu was used to trace the fate of the follicle cells during the female-to-male sex change and the fate of the Sertoli and interstitial cells during the male-to-female sex change. IHC staining was conducted with triplicate sections for each tissue (n = 3–6 tissue samples in each group).

### Apoptotic assay

TUNEL staining was used to analyze the gonadal apoptosis during female-to-male sex change. The fish gonads were fixed with 4% paraformaldehyde in PBS. TUNEL staining was performed according to the manufacture’s protocol (Promega) as described previously [13]. DNase I-treated series slides were used as a positive control.

### Steroid analysis

Plasma E2 (estradiol) and 11-KT (11-ketotestosterone) were extracted with ethyl ether and subsequently measured with an enzyme immunoassay in the Cayman Assay Kits supplied protocol (Cayman).

### Data analysis

All data are expressed as the mean ± SEM. The values were subjected to analysis via one-way ANOVA, followed by a Student-Newman-Keuls multiple test with *P* < 0.05 indicating a significant difference. A Student’s *t*-test was also conducted to compare the significant differences (*P* < 0.05) between two treatments.

## Results

### Bi-directional and reversible sex change in AI/MT-induced maleness after the withdrawal of the orally administered AI/MT-oral administration

Fig 1A is the schematic picture of the histological characteristics (Fig 1B–1J). In the control fish, a central lumen was observed in the 3.5-mo-old fish. The size, sample number and sexual phase during the experimental 1 are summarized in Table 2. All control fish were female (100%) during the experimental period. Juvenile mono-female (7-mo-old fish with oogonia and primary oocytes) were used for the AI/MT-induced masculinization (Fig 1B). Many advanced male germ cells (including spermatocytes and spermatozoa) were observed together with few regressed oocytes (female-to-male sex change) in the fish treated with the orally administered AI for 3 mo (Fig 1C). Three wks after AI-termination, a transitive stage of gonads with many early germ cells was identified in the AI-terminated fish (Table 2). Five wks after AI-termination, newly developed primary oocytes were observed in the AI-terminated fish (Fig 1E, Table 2). Nine wks after AI-termination, many primary oocytes were observed in the AI-terminated fish (male-to-female sex change) (Fig 1F Table 2).

**Table 2:** Sexual phase of fish during the experimental period including 3 mo AI/MT-oral administration and 0–9 weeks of AI/MT-termination.

Administration periods				Sexual phase		
[treatment (months) + termination (weeks)]	Sample No.	Total length (cm)	Body weight (g)	Female	Interphase	Male
Initial control	12	25.5 ± 0.58	280.0 ± 21.11	12	0	0
Control						
3 + 0	8	26.9 ± 1.51	367.4 ± 45.54	8	0	0
3 + 3	3	28.0 ± 1.04	311.9 ± 41.70	3	0	0
3 + 5	3	34.0 ± 1.15	736.5 ± 100.14	3	0	0
3 + 9	3	35.0 ± 1.00	571.6 ± 52.21	3	0	0
AI (20 mg/kg of diet)						
3 + 0	8	27.3 ± 0.58	316.5 ± 24.52	0	0	8
3 + 3	3	28.3 ± 0.88	359.2 ± 34.76	0	2	1
3 + 5	3	34.0 ± 1.15	674.5 ± 25.33	1	2	0
3 + 9	3	33.0 ± 2.08	617.5 ± 14.36	3	0	0
MT (30 mg/kg of diet)						
3 + 0	8	27.4 ± 1.08	324.5 ± 34.76	0	0	8
3 + 3	3	28.3 ± 1.45	359.6 ± 55.73	0	2	1
3 + 5	3	30.0 ± 1.52	460.6 ± 62.25	0	3	0
3 + 9	3	34.7 ± 0.33	600.2 ± 14.18	3	0	0

Female: broad distribution of oocytes without advanced male germ cells (spermatocytes and spermatozoa)

Interphase: broad distribution of early germ cells with few oocytes.

Male: broad distribution of advanced male germ cells with few oocytes (oocyte area smaller than 10%)

The male phase (with many spermatozoa) was observed in the MT-administered fish (female-to-male sex change) after the fish had been treated with the orally administered MT for 3 mo (Fig 1G, Table 2). Three wks and 5 wks after termination of the MT, a transitive stage of gonads with many early germ cells was identified in the MT-terminated fish (Fig 1H and 1I). Nine wks after termination of MT, many primary oocytes were observed in the MT-terminated fish (male-to-female sex change) (Fig 1J).

### Sex steroid levels in plasma are associated with the alternation of sexual fate (bi-directional sex change)

**Fig 2:**
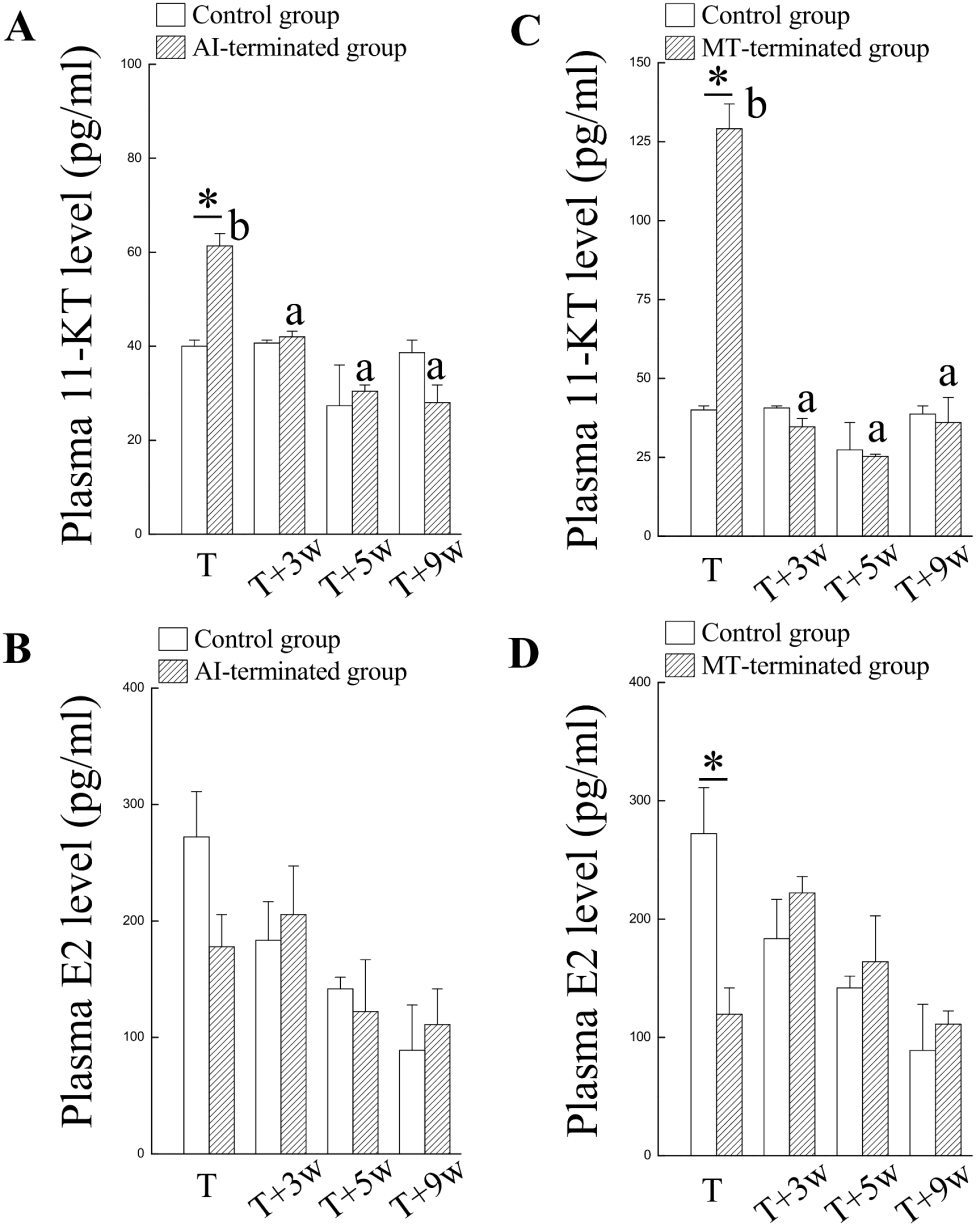
Plasma levels of sex steroids during sexual fate alternation in fish receiving oral administration of AI/MT. The AI/MT-treated fish were functional males after 3 mo oral administration (T). The AI/MT-induced maleness was reversible, and the male-to-female sex change occurred 3 wks after MT-termination (T+3w). (A) and (B) Plasma sex steroids (11-KT and E2) in AI-administered fish and control fish. (C) and (D) Plasma sex steroids (11-KT and E2) in MT-administered fish and control fish. Superscript letters indicate one-way ANOVA and a Student-Newman-Keuls multiple test (*P* < 0.05). Asterisk indicates a Student *t*-test (*P* < 0.05).

Significantly higher levels of plasma 11-KT were identified in the AI-administered fish after 3 mo of oral administration (T) compared with the control fish (Fig 2A). No difference in the plasma 11-KT levels was detected between the two groups (T+3w and control) 3 wks after AI termination (Fig 2A). No difference in the plasma E2 levels was identified between the two groups (AI and control) during experiment periods (Fig 2B). These results indicate that sexual fate alternation (male-to-female) occurred spontaneously in the fish with the AI-induced maleness after AI withdrawal.

Significantly higher levels of plasma 11-KT were identified in the MT-treated fish after 3 mo of oral administration (T) compared with the control fish (Fig 2C, Table 2). No difference in the plasma 11-KT levels was detected between the two groups (T+3w and control) 3 wks after MT termination (Fig 2C). Significantly lower levels of plasma E2 were identified in the MT-treated fish after 3 mo of treatment (T) compared with the control fish (Fig 2D). No difference in the plasma E2 levels was detected between the two groups (T+3w and control) 3 wks after MT termination (Fig 2D). These results indicate that sexual fate alternation (male-to-female) occurred spontaneously in the fish with the MT-induced maleness after withdrawal of the MT.

### The expressions of sex-related genes in the gonad are associated with the alternation of sexual fate (bi-directional sex change)

**Fig 3:**
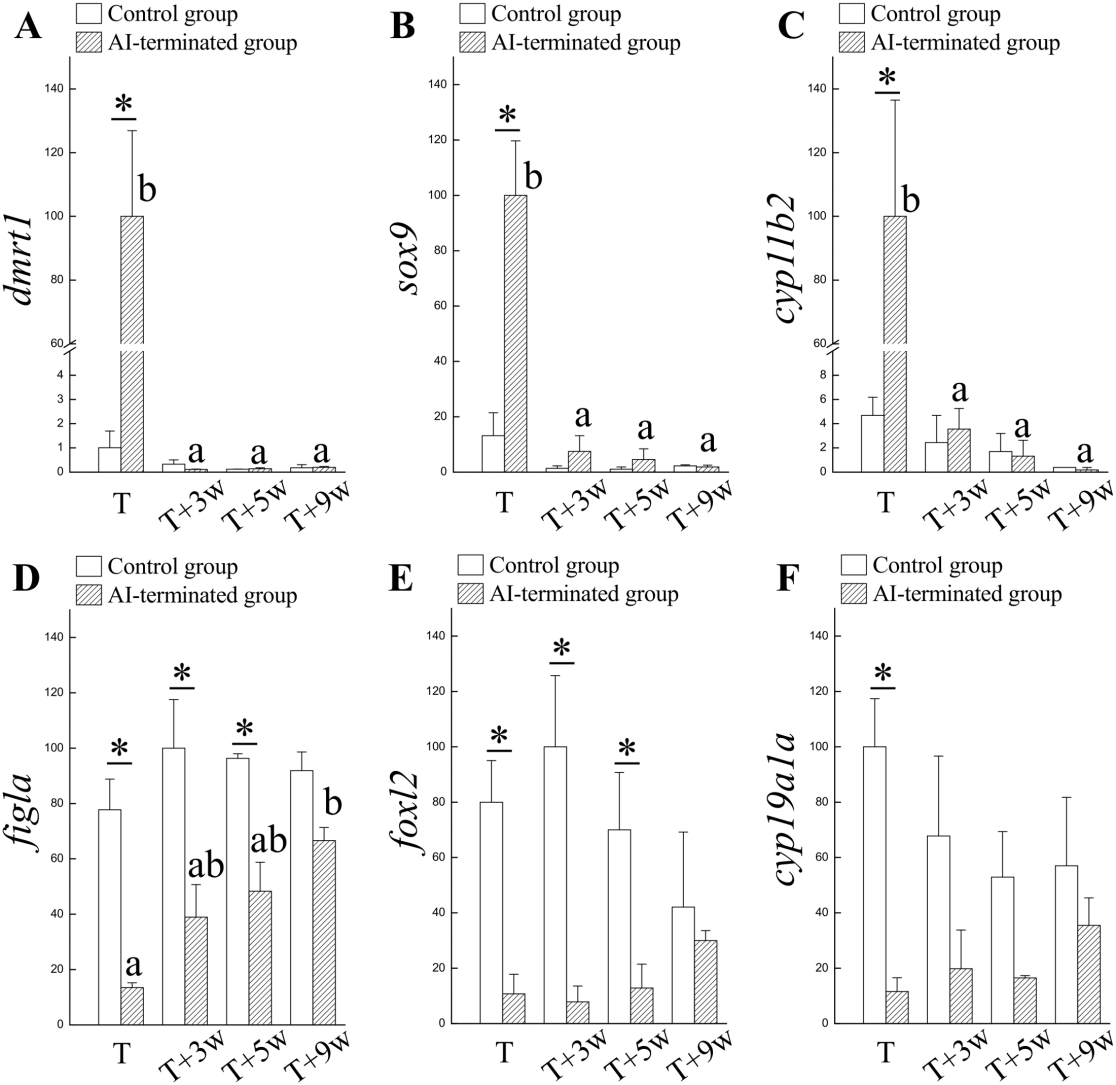
Expression profiles of sex-related genes during sexual fate alternation in fish orally administered AI. The AI-treated fish were functional males after 3 mo oral administration (T). The AI-induced maleness was reversible, and the male-to-female sex change occurred 3 wks after AI-termination (T+3w). (A), (B), and (C) Expression of male-related genes *dmrt1*, *sox9*, and *cyp11b2*, respectively, during the male-to-female sex change that occurred after AI-termination. (D), (E), and (F) Expression of female-related genes *figla*, *foxl2*, and *cyp19a1a*, respectively, during the male-to-female sex change that occurred after AI-termination. Superscript letters indicate one-way ANOVA and a Student-Newman-Keuls multiple test (*P* < 0.05). Asterisk indicates Student *t*-test (*P* < 0.05).

Higher expressions of male-related genes (*dmrt1*, *sox9*, and *cyp11b2*) were identified in maleness (in the AI-administered fish) compared with femaleness (in the control fish) (Fig 3A–3C). Conversely, the female-related genes (*figla*, *foxl2*, and *cyp19a1a*) exhibited a lower expression in maleness (in the AI-administered fish) compared with femaleness (in the control fish) (Fig 3D–3F). The male-related genes in the AI-terminated fish were significantly decreased compared to the levels in the control females 3 wks after AI withdrawal (Fig 3A–3C). Three wks after AI withdrawal, the female-related genes (*figla* and *foxl2*) were maintained at lower levels in the AI-terminated fish (Fig 3D and 3E) compared with the female control fish. No differences in the female-related genes (*cyp19a1a*) were found in the control (femaleness) and AI-terminated fish (at 3 to 9 wks after AI withdrawal) (Fig 3F). The decreased expression in male-related genes was faster than the increase in female-related genes in the AI-terminated fish (Fig 3A–3C).

**Fig 4:**
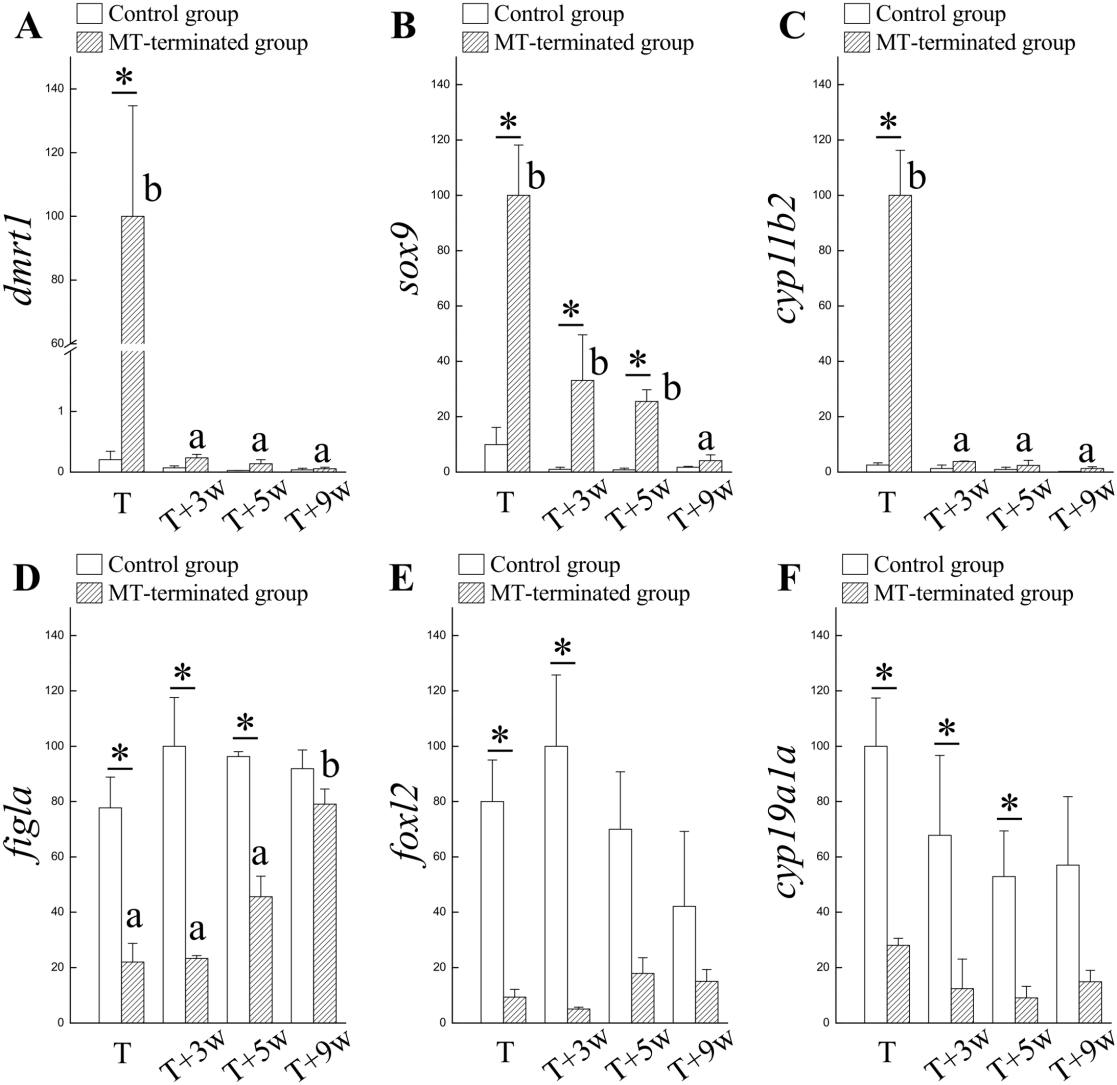
Expression profiles of sex-related genes during sexual fate alternation in fish orally administered MT. The MT-treated fish were functional males after 3 mo oral administration (T). The MT-induced maleness was reversible, and the male-to-female sex change occurred 3 wks after MT-termination (T+3w). (A), (B), and (C) Expression of male-related genes *dmrt1*, *sox9*, and *cyp11b2*, respectively, during the male-to-female sex change that occurred after MT termination. (D), (E), and (F) Expression of female-related genes *figla*, *foxl2*, and *cyp19a1a*, respectively, during the male-to-female sex change that occurred after MT termination. Superscript letters indicate one-way ANOVA and a Student-Newman-Keuls multiple test (*P* < 0.05). Asterisk indicates a Student *t*-test (*P* < 0.05).

Increased expressions of male-related genes (*dmrt1*, *sox9*, and *cyp11b2*) were identified in fish exhibiting maleness (MT-administered fish) compared with fish exhibiting femaleness (control fish) (Fig 4A–4C). Conversely, the female-related genes (*figla*, *foxl2*, and *cyp19a1a*) exhibited a higher expression in femaleness (control fish) compared with maleness (MT-administered fish) (Fig 4D–4F). The male-related genes (*dmrt1* and *cyp11b2*) in the MT-terminated fish were significantly decreased compared to the levels of the control females 3 wks after the MT withdrawal (Fig 4A and 4C). However, *sox9* expression in the MT-terminated fish was maintained at high levels 3 wks and 5 wks after MT withdrawal (Fig 4B). Three wks and 5 wks after the MT withdrawal, the female-related genes (*figla*, *foxl2* and *cyp19a1a*) were maintained at lower levels in the MT-terminated fish compared with control fish (Fig 4D–4F). Except for the *sox9* gene, the decrease in male-related genes was faster than the increase in the female-related genes in the MT-terminated fish (Fig 4A–4F). Based on the results shown in Figs 3 and 4, we suggest that the AI/MT-treated maleness is spontaneously regressed after AI/MT-termination; however, these fish do not immediately enter femaleness.

### Cell replacement for the sexual fate alternation during the sex change

#### Increased cell proliferation of gonadal soma cells in the process of MT-implanted female-to-male sex change

**Fig 5:**
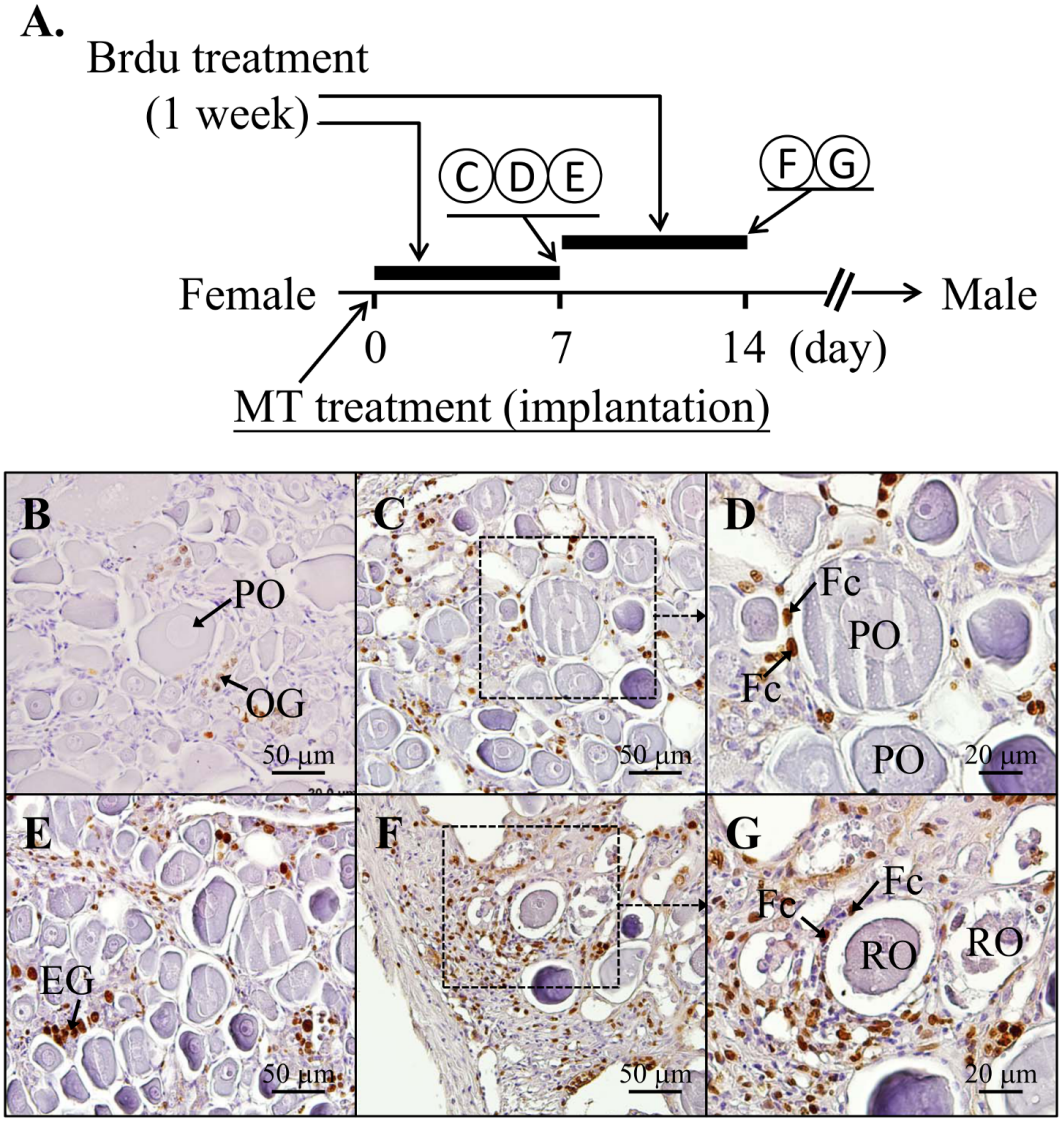
High cell proliferative activity in gonadal soma cells with regressed oocytes in the process of MT-induced female-to-male sex change. (A) Time table of Brdu treatment and the sampling program during the sexual fate alternation from female to male in MT-implanted fish (100 μg MT/mg pellet; 200 mg pellet/kg of body weight). Brdu treatment was given on day 0 and 3 and fish were collected on day 7 of MT-implantation. Brdu treatment was given on day 7 and 10 and fish were collected on day 14 of MT-implantation. The gonadal status was analyzed by IHC staining of Brdu as follows: (B) Initial control female. (C-G) IHC staining of Brdu showed broadly signals in the gonadal soma cells during the female-to-male sex change. (C) and (D) are the samples collected 7 days after MT-implantation. (F) and (G) are the samples collected after 14 days of MT-implantation. (D) and (G) High magnitude pictures of Fig C and G showed broad Brdu signals in the soma cells in the presence of regressed oocytes. EG, early germ cells; PO, primary oocyte; OG, oogonia; RO, regressed oocyte; Fc, follicle cells.

**Fig 6:**
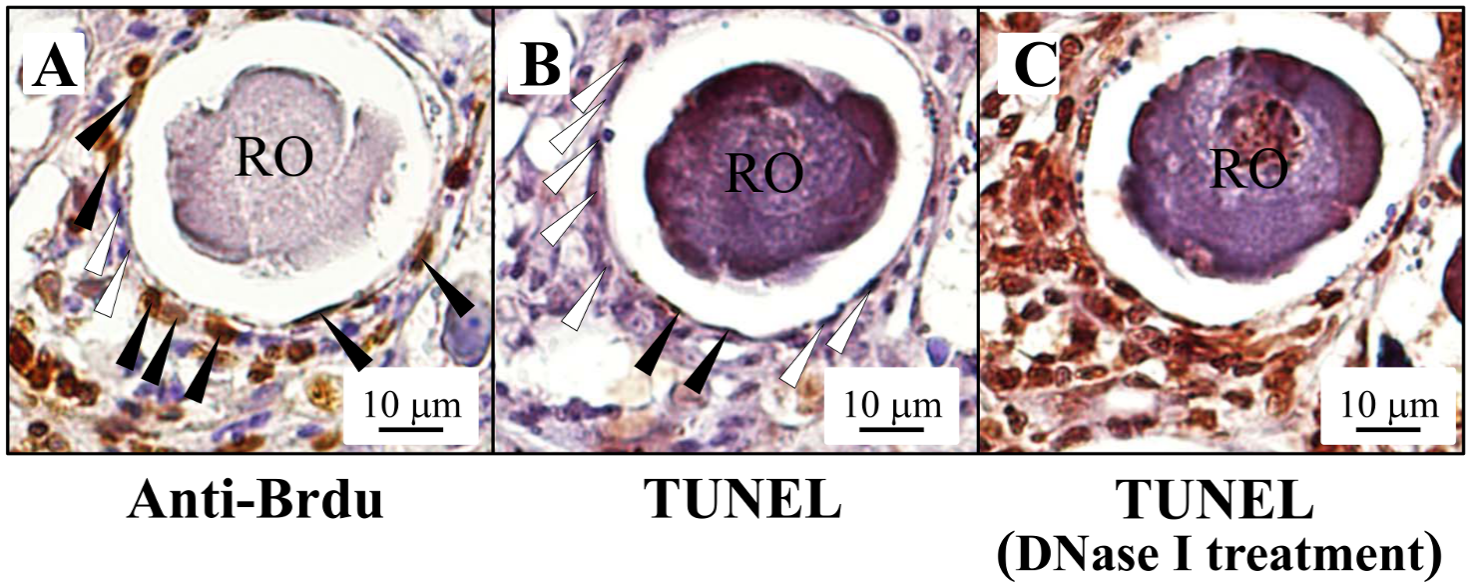
High survival and proliferative activity in the surrounding gonadal soma cells during female-to-male sex change. Gonadal cell status was analyzed by proliferative activity (Brdu treatment) and TUNEL assay. (A) IHC staining of Brdu showed broad signals (with solid arrowheads) in the regressed oocyte-surrounding somatic cells. A hollow arrowhead indicates the cells with no Brdu-stainings. (B) Apoptotic analysis revealed slight signals in the regressed oocyte-surrounding cells (with solid arrowheads). The hollow arrowhead indicates the cells with no TUNEL staining signals. (C) In contrast, the DNase I treatment in the serial sections resulted in robust and broad TUNEL staining signals. RO, regressed oocyte.

To clarify the precise process of sexual fate alternation, the changes in the soma cells (follicle cells in females and Sertoli cells in males) are important. Brdu was given to identify the divided cells during oocyte depletion in the MT-implanted masculinization. Fig 5A shows the schematic design of the experimental procedures. In the control fish, most Brdu-incorporated cells were oogonia and not the somatic cells (Fig 5B). However, according to the IHC staining of Brdu, our results indicate that the primary oocyte-surrounding somatic cells (Fig 5C and 5D) and the regressed oocyte-surrounding somatic cells (Fig 5F and 5G) have increased proliferating activity in the MT-treated fish compared with the control fish (Fig 5B). To confirm the proliferative activity of the regressed oocyte-surrounding somatic cells (Fig 6A), a TUNEL assay was used to identify the cells undergoing apoptosis while the MT implantation was inducing the regression of the oocytes. Only slightly apoptotic signals were observed in the regressed oocyte-surrounding somatic cells (Fig 6B) compared with DNase I treatment in the serial sections which showed broad and robust apoptotic signals (Fig 6C). These results reveal that depleted oocyte-surrounding somatic cells could proliferate and survive well during the female-to-male sex change.

#### Alternation of sexual fate of gonadal soma cells in the MT-implanted female-to-male sex-changed fish

**Fig 7:**
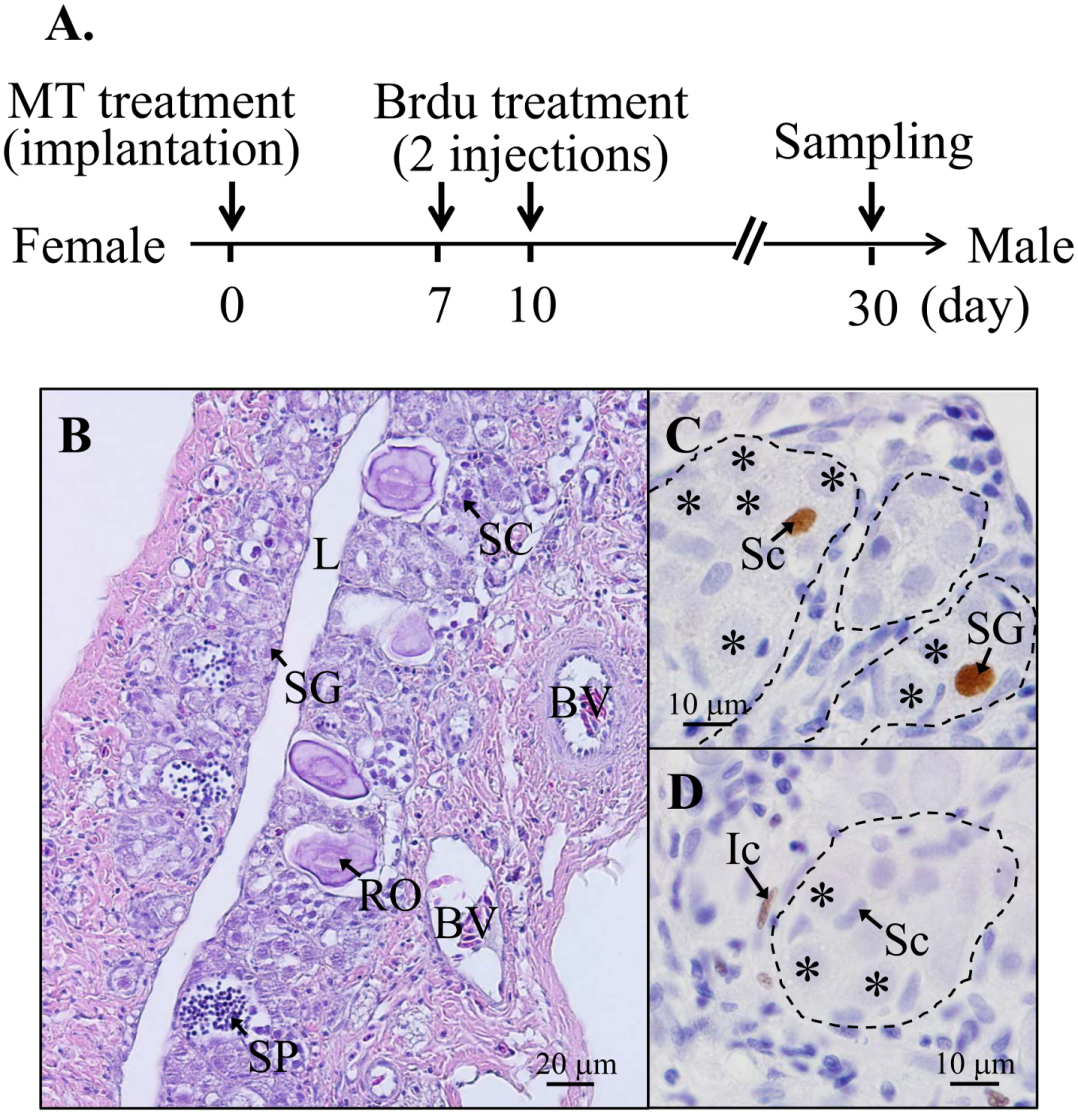
The sexual fates of gonadal soma cells during female-to-male sex change. (A) Time table of Brdu treatment (given on days 7 and 10) in the process of MT-implanted female-to-male sex change. The fish were collected on day 30. (B) MT-implantation (100 μg MT/mg pellet; 200 mg pellet/kg of body weight) was given for 30 days. (C) and (D) IHC analysis of a gonad with the Brdu antibody. SG, spermatogonia; SC, spermatocyte; SP, spermatozoa; L, central lumen; BV, blood vessel; Sc, Sertoli cell; Ic, interstitial cell. An asterisk indicates the spermatogonia.

Brdu was used to trace the sexual fate of the oocyte-depleted follicle cells during the female-to-male sex change during the MT-implanted masculinization. Fig 7A show the schematic design of the experimental procedures. Many spermatogonia with a small portion of advanced male germ cells (including spermatocytes and spermatozoa) were observed together with few regressed oocytes (female-to-male sex change) in the fish treated with MT implantation for 1 mo (Fig 7B). Brdu-incorporated cells were identified in the surrounding soma cells of the spermatogonia, including the Sertoli cells (Fig 7C) and interstitial cells (Fig 7D) in the MT-induced male. Taken together, oocyte-depleted somatic cells with increased proliferating activities (somatic cells with Brdu-incorporation in Fig 5C–5G) indicate that a portion of the male somatic cells is coming from the oocyte-depleted somatic cells (Fig 7C and 7D). Thus, our results reveal that oocyte-depleted somatic cells may transdifferentiate to Sertoli cells and interstitial cells in the MT-treated male.

#### Arrested proliferative activity of the gonadal soma cells in the MT-terminated male-to-female reversible sex-changed fish

**Fig 8:**
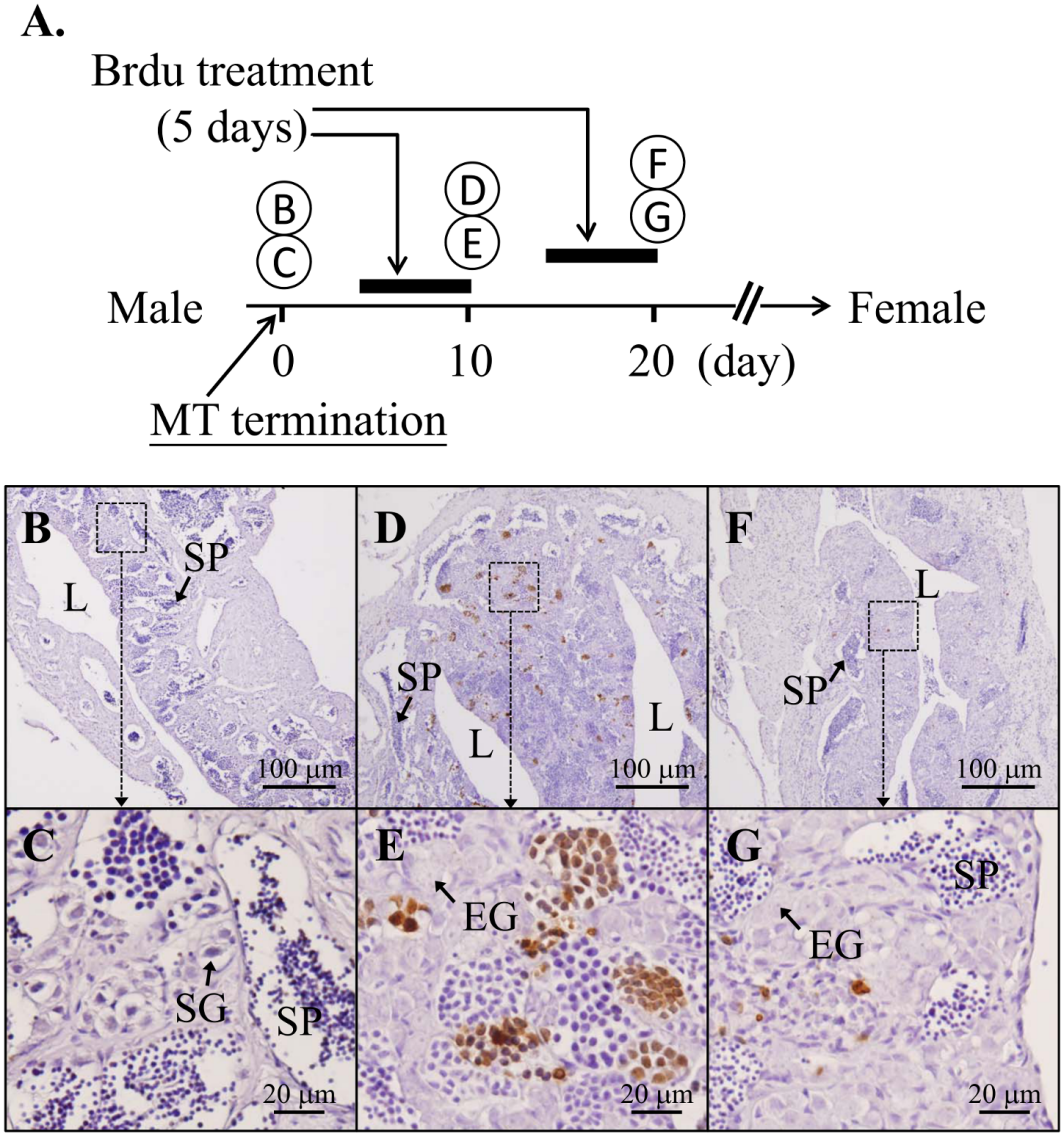
The sexual fates of gonadal soma cells during male-to-female sex change. (A) Time table of Brdu treatment: the fish treated on days 6 and 8 and collected on day 10; and the fish treated on days 16 and 18 and collected on day 20 in the male-to-female sex change in the MT-terminated fish. The fish was already induced to maleness with 3 mo of orally administered MT (30 mg/kg of diet). (B-G) IHC analysis of a gonad with the Brdu antibody. (B) MT-induced male after 3 mo of orally administered MT. (C) The high magnification of Fig B. (D) Male-to-female sex change was occurred in the MT-terminated fish 10 day after termination of MT and showed high proliferation in the advanced germ cells. (E) The high magnification of Fig D. (F) Male-to-female sex change occurred in the MT-terminated fish 20 days after MT (without Brdu-stainings in the gonad soma cells and early germ cells). (G) The high magnification of figure F. L, central lumen; EG, early germ cells; SP, spermatozoa.

Brdu was applied to trace the sexual fate of male somatic cells during the male-to-female sex change in the MT-terminated fish. Fig 8A show the schematic design of the experimental procedures. MT-induced males (10-mo-old fish with advanced male germ cells) after 3 mo of orally administered MT were used to trace the male soma fate during the male-to-female sex change (Fig 8B and 8C). All MT-terminated fish were female (male-to-female sex change) 2 mo after withdrawal of the MT. Advanced male germ cells showed increased proliferating activities (with Brdu-incorporation), but the early germ cells were in dormancy (without Brdu-incorporation) following the Brdu-treatment during the periods of 6- to 10-days (Fig 8D and 8E) and 16- to 20-days after termination of the MT (Fig 8F and 8G). Absence of Brdu-incorporation was identified in the gonadal soma cells during the process of the male-to-female sex change (Fig 8D–8G). There were no Brdu signals in the testis without Brdu treatment (data not shown). In addition, no Brdu-incorporated oocyte-surrounding cells were observed in the MT-terminated fish 2 mo after withdrawal of MT (Brdu was given on day 6 and day 8 after MT-termination; data not shown).

## Discussion

The treatment of sexually undifferentiated fish by the administration of sex steroids has been demonstrated to work well in a wide range of gonochoristic fish. However, sex steroid treatment is rarely successfully administered during the stable sexual phase in hermaphroditic species. In this study, the oral administration of both AI/MT for 3 mo or the implantation of MT for one mo could make the female grouper undergo a sex change to a male. We demonstrate that the sex of the juvenile grouper is returned to femaleness even if the fish have undergone AI/MT-induced masculinization. Furthermore, maleness instantaneously began to regress after withdrawal of the fish from AI/MT. In addition, the process of soma cell replacement was different between the processes of the female-to-male and male-to-female sex changes.

### Sex steroids are important in the regulation of sexual fate in protogynous grouper

In the present study, we demonstrate that AI-/MT-stimulated androgen elevation resulted in masculinization with high 11-KT levels in juvenile grouper. E2 levels were reduced in MT-treated fish, whereas no change was observed in the AI-treated fish. Furthermore, the reduced 11-KT levels and increased E2 levels accompanied a rapid male-to-female sex change after AI-/MT-termination. Low E2 levels and a concomitant increase in the T and 11-KT levels have been correlated with the female-to-male sex change in the protogynous honeycomb grouper [27] and protogynous Ballan wrasse [28]. Furthermore, the induction of maleness with AI was completely blocked by co-treatment with E2 in female grouper [29]. Conversely, low 11-KT levels via a concomitant increase in the E2 levels were correlated with the male-to-female sex change in the protandrous black porgy [5]. In addition, in the black porgy, the treatment with AI induced significantly increased levels of plasma 11-KT and completely blocked the natural sex change in black porgy [30]. Taken together, our data demonstrate that sex steroids are involved in the sexual phase of hermaphroditic teleosts [3].

### A rapid process for the reversible sex change in grouper

In the present study, we demonstrate that AI- and MT-induced masculinization is reversed from male to female after AI/MT-termination in juvenile grouper. We also demonstrated for the first time the bi-directional sex change in a rapid process with respect to gonadal histology, the changes of gene expression and sex steroid levels. Unlike most vertebrates, sex determination resulted from an initial switch of either at testis-differentiating or an ovary-differentiating molecular cascade in an exclusive manner in fish [1, 31]. In hermaphroditic fish, the sex was determined in the initial gonadal differentiation (primary sex determination) and affected by the stability of the sexual phase (secondary sex determination) [13]. In protogynous fish, steroid-induced males are transient and reversible in sequential sex change [20, 23, 32]. Female development also could not be maintained in the E2-induced female (sex is reversed from female to male) after E2 termination in the protandrous black porgy [30, 33, 34, 35]. Thus, hermaphroditic fish regulate both sexes at the same time; that is one sex develops and another sex regresses.

The sexual fate decision (secondary sex determination) in hermaphroditic fish is dependent on various parameters, including age, body size, and social factors [1]. In wrasse fish [36, 37] and dusky anemone fish [38, 39], the territorial male had an increased Gnrh neuron size and number compared with the female. In the wrasse fish, the female-to-male sex change was stimulated by gonadotropin treatment [40]. Furthermore, sex change in the gobiid fish is mediated through a rapid switch of the gonadotropin receptor [41]. Natural sex change in the black porgy from a male to female is correlated with the lack of Lh peaks in the non-spawning season [42]. In the honeycomb grouper [43], the female-to-male sex change was stimulated by gonadotropin treatment. Taken together, these data suggest that the secondary sex determination is controlled by the brain-pituitary-gonad axis. Our present data also demonstrate that AI/MT treatment could induce sex change; however, it is not a stable sexual phase, and the maintenance of the second sexual phase (secondary sex determination) is associated with sex steroids.

### The sex-related gene characteristics are associated with male and female sex change

In the present study, we demonstrate that male-related gene (*sox9*, *dmrt1*, and *cyp11b2*) expressions are significantly decreased after AI/MT-termination. In grouper, *sox9* expression is a male marker and only expressed in Sertoli cells [44]. In addition, grouper *dmrt1* is not only differentially expressed in different stage gonads but is also restricted to the specific stages and specific cells of spermatogenesis [44]. The grouper Dmrt1 protein exists only in spermatogonia, primary spermatocytes and secondary spermatocytes [44]. Conversely, the key enzyme for 11-KT production, Cyp11b2, exhibited strong signals in the grouper interstitial cells [45]. Taken together, our results demonstrate that AI/MT-induced masculinization is a transient state, and male characteristics (including *sox9*, *dmrt1*, and *cyp11b2* expression) are dramatically reduced after AI/MT-termination (male-to-female sex reversal).

In contrast to the rapid decreases in the male-related genes, our results demonstrate that the female-related genes (*figla*, *foxl2*, and *cyp19a1a*) were gradually increased (but not rapidly) after AI/MT-termination. In fish, *figla* is a female marker expressed in the ovary [24, 46]. In addition, black porgy *figla*/Figla existed only in oocytes, but not oogonia [47]. Conversely, grouper *foxl2* and *cyp19a1a* expression levels were significantly down regulated from the transitional phase to the completion of the female-to-male sex change [48, 49]. In grouper, *cyp19a1a*/Cyp19a1a is not only a female marker that is only expressed in follicle cells but also the enzyme for estrogen production [49]. Our results demonstrate that the rapidly reduced male characteristics did not accompany a rapid increase in female characteristics. Conversely, in the *in vitro* experiment with grouper ovarian fragments, MT had no direct effects on the expression of *cyp19a1a* or the activity of gonadal aromatase [50]. We suggest that the sexual phase may be mediated by the upstream regulation of the brain-pituitary-gonad axis.

### An active gonad with high proliferation, functional alternation and replacement of gonadal soma cells during sex change from female-to-male

In the present study, we find that oocyte-depleted somatic cells with strong proliferative activity could survive well during a female-to-male sex change. We further demonstrate that the oocyte-depleted follicle cells immediately altered the function of soma cells from female to male in the newly generated male gonad. Similar to our present study, oocyte-depleted follicle cells alter the sexual fate from female to male function during the female-to-male sex change in protogynous wrasse fish [14]. In black porgy, ectopic located oocytes in regenerated testes can alter the sexual fate of gonadal soma cells from male to female function [13]. In mammals, oocyte-depleted follicle cells subsequently acquire the morphological and genetic characteristics of Sertoli cells [51]. Conversely, in medaka [8] and zebrafish [9], germ cell (female or male)-deficient fish typically develop a phenotypic male. Therefore, our data suggest that the transdifferentiation of soma cells from the female to male sexual phase occurs in groupers. Taken together, these data also indicate that female germ cells may play an important role in the maintenance of follicle cell function in groupers.

### A dormant gonad with inactive early germ cells and non-proliferative activity of gonadal soma cells during the reversible sex change from male-to-female

A delayed increase of female-related gene expression (Figs 3 and 4) and dormant early germ cells suggest that there is a transitive and dormant stage in gonads during the reversible sex change from male to female. Thus, the dormant gonads in the transitive stage (without male and female characteristics) are waiting for the appropriate signals to regenerate the female environment. Taken together, these results indicate that AI/MT-terminated fish did not have appropriate female environmental conditions. Therefore, even male characteristics were reduced in the dormant gonad, which still requires more time for the reconstruction of the appropriate female environment (oocyte regeneration).

Our data also demonstrate that Sertoli cells and interstitial cells show low proliferating activities (no Brdu-incorporation) during the male-to-female sex change. Conversely, advanced male germ cells showed increased proliferating activities, whereas the early germ cells were in a stage of dormancy after MT termination. Therefore, in contrast to the female-to-male sex change, our present data demonstrated that follicle cells do not derive from male soma cells in the male-to-female sex change because there is no Brdu-incorporation in the male soma cells. In fish, the sexual fate of early germ cells was flexible during development becoming either early spermatogonia or oogonia, and sex was dependent on the gonadal sex [52]. The soma cells in the male-to-female sex change were not derived from the Sertoli cells and interstitial cells. The source of those new soma cells in the male-to-female sex change grouper is still not clear. A transdifferentiation of soma cells from a male-to-female sexual fate was found in black porgy [12]. Our data demonstrate the different mechanisms between black porgy and grouper in the sex change from male-to-female although they are both hermaphroditic fish.

### Conclusion

We demonstrate for the first time that AI/MT-induced masculinization (female-to-male) is a transient state and that a reversible sex change (male-to-female) is initiated instantaneously with termination of AI/MT in the orange-spotted grouper. Our data also indicate for the first time that the grouper applies different approaches to replace the gonad soma cells from one sex to another in the bi-directional sex change. In the female-to-male sex change, oocyte-depleted follicle cells alter their function from a female (follicle cells) to male (Sertoli cells) sexual fate (transdifferentiation) during the AI/MT-induced female-to-male sex change. In contrast, in the male-to-female sex change, the advanced male germ cells were depleted, male soma cells were not proliferative and the male soma cells did not alter their function from male to female after AI/MT-termination (male-to-female sex change). Early germ cells were in the dormancy stage and appeared in the transitive gonad in the process of male-to-female sex change. The source of follicle cells was not clear in the male-to-female sex change grouper. The changes in the gonadal soma sexual fate were correlated with the plasma levels of endogenous sex steroids and the gonad expression of sex-related genes.

## Supporting Information

S1 Fig Histological analysis of the gonad after termination of MT. (A) The gonadal morphology of the orange-spotted grouper 2 wks after termination of MT and the schematic picture of the histological characteristics (Figs B–G). (B), (C) and (D) The gonad status at the anterior part, middle part and posterior part, respectively. (E), (F) and (G) are the high magnified pictures of Figs B, C and D, respectively. L, central lumen; EG, early germ cell; SC, spermatocyte; SP, spermatozoa; PO, primary oocyte.
(TIF)
