# Supplementary figures and images for: Sexual Fate Reprogramming in the Steroid-Induced Bi-Directional Sex Change in the Protogynous Orange-Spotted Grouper, Epinephelus coioides
Source: PLoS One. 2015 Dec 29;10(12):e0145438. doi: 10.1371/journal.pone.0145438 (PMC4694621; doi:10.1371/journal.pone.0145438)

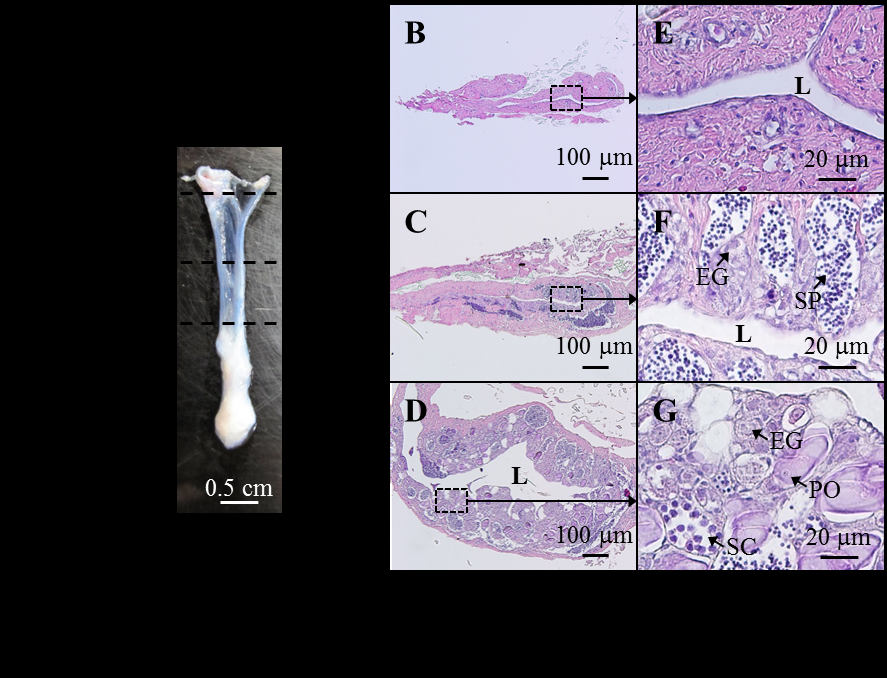

Supplement: S1 Fig — (A) The gonadal morphology of the orange-spotted grouper 2 wks after termination of MT and the schematic picture of the histological characteristics (Figs B–G). (B), (C) and (D) The gonad status at the anterior part, middle part and posterior part, respectively. (E), (F) and (G) are the high magnified pictures of Figs B, C and D, respectively. L, central lumen; EG, early germ cell; SC, spermatocyte; SP, spermatozoa; PO, primary oocyte. (TIF) [file pone.0145438.s001.tif]
